# Supplementary material for: Eastern Grey Kangaroo (Macropus giganteus) Vigilance Behaviour Varies between Human-Modified and Natural Environments
Source: Animals (Basel). 2019 Jul 27;9(8):494. doi: 10.3390/ani9080494 (PMC6719249; doi:10.3390/ani9080494)
Supplement: Supplementary file 1 [file animals-09-00494-s001.zip › Animal Supporting Documents Final.docx]

# Supporting Documents

## Supporting documents 1: study site locations and images

| Table 1: Site locations across South-East Queensland and the Australian Capital Territory. Urban sites are shown in grey, non-urban sites are shown in white. | | | | | | |
| --- | --- | --- | --- | --- | --- | --- |
| Site | **Region** | **Latitude** | **Longitude** | **Human Population Density**  **(persons/km^2)^** | **Observed Mob Size** | |
|  |  |  |  |  | **Summer** | **Winter** |
| Weston Park | ACT | 35°17'28.75"S | 149° 5'34.59"E | 408.2 | 41-50 | 21-30 |
| Red Hill | ACT | 35°20'9.33"S | 149° 7'15.86"E | 673.6 | 200+ | 31-40 |
| Mount Taylor | ACT | 35°21'45.87"S | 149° 4'10.41"E | 1671.6 | 31-40 | 21-30 |
| Farrer Ridge | ACT | 35°23'9.71"S | 149° 6'25.47"E | 1641.5 | 21-30 | 31-40 |
| South Lawson | ACT | 35°13'26.10"S | 149° 5'53.55"E | 1562.7 | 31-40 | 31-40 |
| Gold Creek | ACT | 35°11'15.23"S | 149° 5'6.31"E | 1026.4 | 101-150 | 101-150 |
| Rendezvous Creek | ACT | 35°43'56.42"S | 148°59'43.44"E | 0 | 21-30 | 21-30 |
| Mount Clear | ACT | 35°52'7.82"S | 149° 0'51.71"E | 0 | 31-40 | 41-50 |
| Gudgenby | ACT | 35°44'37.75"S | 148°59'19.86"E | 0 | 51-100 | 11-20 |
| Nass Valley Flats | ACT | 35°51'22.05"S | 148°59'24.94"E | 0 | 31-40 | 51-100 |
| Tidbinbilla | ACT | 35°26'23.65"S | 148°56'40.84"E | 11 | 200+ | 51-100 |
| Googong | ACT | 35°25'45.73"S | 149°15'21.80"E | 5.7 | 41-50 | 11-20 |
| Wacol | SEQ | 27°33'56.58"S | 152°55'32.25"E | 327.7 | 200+ | 200+ |
| Park Ridge | SEQ | 27°42'12.65"S | 153° 2'10.86"E | 395.4 | 21-30 | 0-10 |
| Twin Waters | SEQ | 26°37'29.00"S | 153° 5'12.52"E | 342.7 | 11-20 | 11-20 |
| Little Mountain | SEQ | 26°46'33.63"S | 153° 6'9.95"E | 290.7 | 0-10 | 0-10 |
| Sippy Downs | SEQ | 26°43'8.74"S | 153° 3'52.74"E | 722.9 | 11-20 | 0-10 |
| Coolum | SEQ | 26°33'2.26"S | 153° 5'25.59"E | 836.7 | 0-10 | 0-10 |
| Mount Walker | SEQ | 27°40'13.69"S | 152°36'28.22"E | 17.9 | 31-40 | 41-50 |
| Allenview | SEQ | 27°52'33.70"S | 152°56'11.66"E | 8.7 | 0-10 | 0-10 |
| Elanda Point | SEQ | 26°15'10.95"S | 152°59'53.16"E | 27.6 | 11-20 | 11-20 |
| Toorbul | SEQ | 27° 2'31.28"S | 153° 6'14.10"E | 143.5 | 21-30 | 11-20 |
| Landsborough | SEQ | 26°49'36.51"S | 152°58'38.30"E | 77.8 | 21-30 | 11-20 |
| Weyba Downs | SEQ | 26°26'16.05"S | 153° 3'45.67"E | 27.6 | 21-30 | 0-10 |


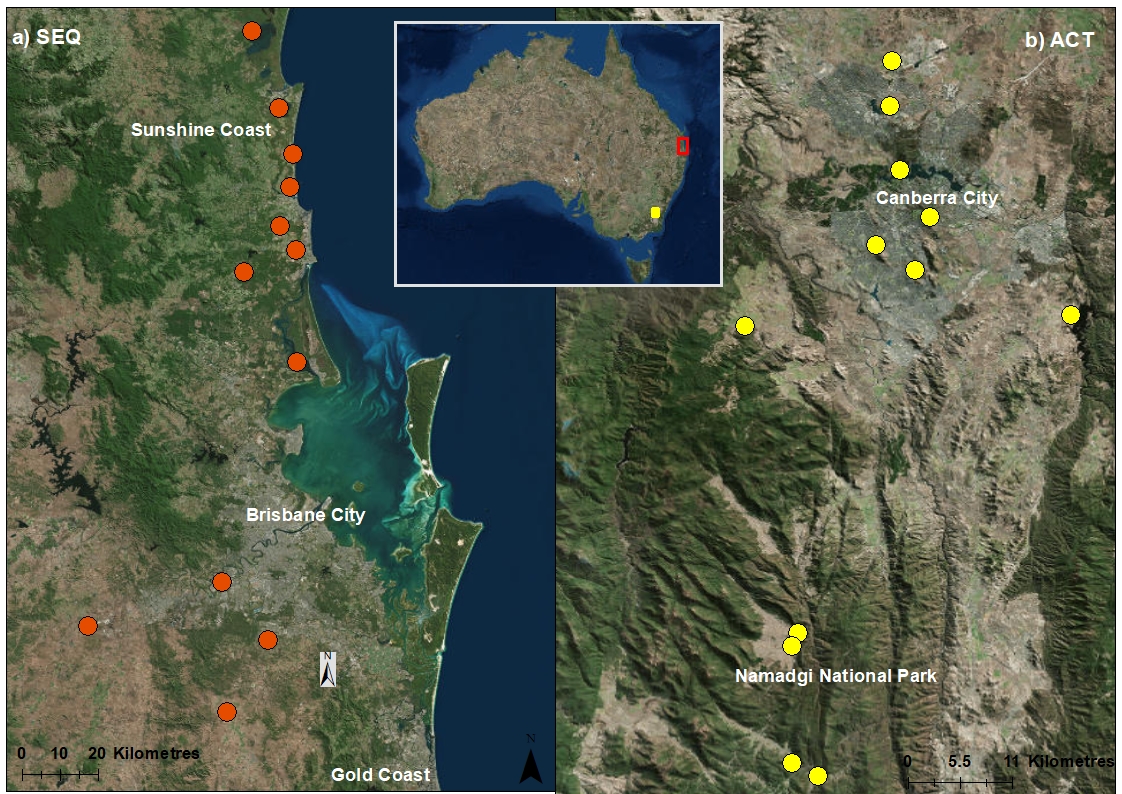


**Fig S1:** Geographic location of the study sites used across two regions in Australia.

## Supporting documents 2: model selection tables

| **Table S1:** Model selection table for the variables influencing **time spent vigilant**. Factors included in each model are denoted by the symbol –, an asterisk denotes interaction terms between variable. HPD (human population density), DTC (distance to cover). The full model and final model with the smallest AICc value are highlighted in bold, with these results being reported in full in table 2. | | | | | | | | | | | |
| --- | --- | --- | --- | --- | --- | --- | --- | --- | --- | --- | --- |
| **Model ID** | **intercept** | **Mob size** | **Sex** | **Land use** | **Rainfall (mm)** | **HPD** | **DTC** | **Season***  **Region** | **df** | **AICc** | **ΔAICc** |
| **m4** | **12.23** |  | **-** |  |  |  | **-** | **-** | **8** | **1727.4** | **0.0** |
| m3 | 11.71 |  | - |  |  | - | - | - | 9 | 1727.7 | 0.3 |
| m5 | 11.35 |  | - |  |  |  |  | - | 7 | 1729.0 | 1.6 |
| m2 | 12.02 |  | - | - |  | - | - | - | 10 | 1729.2 | 1.8 |
| m1 | 11.93 |  | - | - | - | - | - | - | 11 | 1730.7 | 3.3 |
| **m0** | **12.19** | **-** | **-** | **-** | **-** | **-** | **-** | **-** | **12** | **1732.0** | **4.6** |

| **Table S2:** Values from the LMM models highlighted in table 1 as being the final models used for predicting which factors influenced the **time spent vigilant**. (Site was included as a random effect in each model, with the variance (s.d.) being 4.17 (2.04) in the full model and 4.49 (2.12) in model 4. Significant values are shown in bold. | | | | |
| --- | --- | --- | --- | --- |
| **Variable** | **Estimate** | **s.e.** | ***t­*-value** | ***p*-value** |
| *m0 summary* | | | | |
| **Intercept** | **12.20** | **1.27** | **9.60** | **<0.001** |
| Mob size | -0.12 | 0.23 | -0.52 | 0.61 |
| Sex | | | | |
| **Male** | **-1.50** | **0.57** | **-2.61** | **<0.05** |
| Female | 0.0 | 0.0 |  |  |
| Land use | | | | |
| Urban | -0.90 | 1.63 | -0.55 | 0.59 |
| Non-urban | 0.0 | 0.0 |  |  |
| Rainfall (mm) | 0.01 | 0.02 | 0.72 | 0.47 |
| Human Population Density | 0.00 | 0.00 | 1.07 | 0.30 |
| Distance to Cover | -0.44 | 0.39 | -1.13 | 0.26 |
| Season | | | | |
| Winter | -0.06 | 0.81 | -0.08 | 0.94 |
| Summer | 0.0 | 0.0 |  |  |
| Region | | | | |
| ACT | -1.32 | 1.42 | -0.93 | 0.36 |
| SEQ | 0.0 | 0.0 |  |  |
| **Season*Region** | **3.94** | **1.28** | **3.07** | **<0.05** |
| *m4 summary* | | | | |
| **Intercept** | **12.23** | **1.00** | **12.28** | **<0.001** |
| Sex | | | | |
| **Male** | **-1.51** | **0.57** | **-2.65** | **<0.05** |
| Female | 0.0 | 0.0 |  |  |
| Distance to Cover | -0.67 | 0.36 | -1.86 | 0.07 |
| Season | | | | |
| Winter | -0.10 | 0.80 | -0.13 | 0.90 |
| Summer | 0.0 | 0.0 |  |  |
| Region | | | | |
| ACT | -0.97 | 1.18 | -0.82 | 0.42 |
| SEQ | 0.0 | 0.0 |  |  |
| **Season*Region** | **4.48** | **1.17** | **3.83** | **<0.001** |

| **Table S3:** Model selection table for the variables influencing **time spent vigilant in SEQ**. Factors included in each model are denoted by the symbol –, an asterisk denotes interaction terms between variable. HPD (human population density), DTC (distance to cover). The full model and final model with the smallest AICc value are highlighted in bold, with these results being reported in full in table 4. | | | | | | | | | | | |
| --- | --- | --- | --- | --- | --- | --- | --- | --- | --- | --- | --- |
| **Model ID** | **intercept** | **Mob size** | **Sex** | **Land use** | **Rainfall**  **(mm)** | **HPD16** | **DTC** | **Season** | **df** | **AICc** | **ΔAICc** |
| **seqm4** | **13.85** |  | **-** |  | **-** |  | **-** |  | **6** | **824.1** | **0.0** |
| seqm3 | 14.47 | - | - |  | - |  | - |  | 7 | 824.1 | 0.0 |
| seqm2 | 15.32 | - | - |  | - |  | - | - | 8 | 824.1 | 0.0 |
| seqm5 | 13.48 |  | - |  |  |  | - |  | 5 | 825.7 | 1.6 |
| seqm1 | 15.40 | - | - | - | - |  | - | - | 9 | 825.9 | 1.8 |
| **seqm0** | **15.28** | **-** | **-** | **-** | **-** | **-** | **-** | **-** | **10** | **827.9** | **3.8** |

| **Table S4:** Values from the LMM models highlighted in table 3 as being the final models used for predicting which factors influenced the **time spent vigilant in SEQ**. (Site was included as a random effect in each model, with the variance (s.d.) being 2.06 (1.44) in the full model and 2.33 (1.53) in model 4. Significant values are shown in bold. | | | | |
| --- | --- | --- | --- | --- |
| **Variable** | **Estimate** | **s.e.** | ***t­*-value** | ***p*-value** |
| *seqm0 summary* | | | | |
| **Intercept** | **15.28** | **1.27** | **12.03** | **<0.001** |
| Mob size | -0.29 | 0.27 | -1.07 | 0.31 |
| Sex | | | | |
| **Male** | **-3.45** | **0.73** | **-4.75** | **<0.001** |
| Female | 0.0 | 0.0 |  |  |
| Land use | | | | |
| Urban | -0.85 | 2.02 | -0.42 | 0.69 |
| Non-urban | 0.0 | 0.0 |  |  |
| Rainfall (mm) | -0.14 | 0.02 | 0.72 | 0.47 |
| Human population density | 0.00 | 0.00 | 0.23 | 0.82 |
| **Distance to Cover** | **-1.06** | **0.47** | **-2.23** | **<0.05** |
| Season | | | | |
| Winter | -0.95 | 0.78 | -1.22 | 0.23 |
| Summer | 0.0 | 0.0 |  |  |
| *seqm4 summary* | | | | |
| **Intercept** | **13.85** | **0.86** | **16.12** | **<0.001** |
| Sex | | | | |
| **Male** | **-3.46** | **0.72** | **-4.80** | **<0.001** |
| Female | 0.0 | 0.0 |  |  |
| Rainfall (mm) | -0.12 | 0.06 | -1.89 | 0.06 |
| **Distance to Cover** | **-1.12** | **0.44** | **-2.54** | **<0.05** |

| **Table S5:** Model selection table for the variables influencing **time spent vigilant in ACT**. Factors included in each model are denoted by the symbol –, an asterisk denotes interaction terms between variable. HPD (human population density), DTC (distance to cover). The full model and final model with the smallest AICc value are highlighted in bold, with these results being reported in full in table 6. | | | | | | | | | | | |
| --- | --- | --- | --- | --- | --- | --- | --- | --- | --- | --- | --- |
| **Model ID** | **intercept** | **Mob size** | **Sex** | **Land use** | **Rainfall (mm)** | **HPD** | **DTC** | **Season** | **df** | **AICc** | **ΔAICc** |
| **actm5** | **8.06** |  |  | **-** |  |  |  | **-** | **5** | **884.1** | **0.0** |
| actm4 | 8.00 |  |  | - | - |  |  | - | 6 | 884.3 | 0.2 |
| actm3 | 7.04 |  |  | **-** | **-** |  | **-** | **-** | 7 | 885.7 | 1.6 |
| actm6 | 9.68 |  |  |  |  |  |  | **-** | 4 | 886.7 | 2.6 |
| actm2 | 6.75 |  | **-** | **-** | **-** |  | **-** | **-** | 8 | 887.4 | 3.3 |
| actm1 | 7.58 | **-** | **-** | **-** | **-** |  | **-** | **-** | 9 | 888.6 | 4.5 |
| **actm0** | **7.06** | **-** | **-** | **-** | **-** | **-** | **-** | **-** | **10** | **890.6** | **6.5** |

| **Table S6:** Values from the LMM models highlighted in table 5 as being the final models used for predicting which factors influenced the **time spent vigilant in ACT**. (Site was included as a random effect in each model, with the variance (s.d.) being 5.09 (2.26) in the full model and 4.65 (2.16) in model 5. Significant values are shown in bold. | | | | |
| --- | --- | --- | --- | --- |
| **Variable** | **Estimate** | **s.e.** | ***t­*-value** | ***p*-value** |
| *actm0 summary* | | | | |
| **Intercept** | **7.06** | **2.47** | **2.87** | **<0.05** |
| Mob size | 0.08 | 0.34 | -0.24 | 0.81 |
| Sex | | | | |
| Male | 0.49 | 0.85 | 0.57 | 0.57 |
| Female | 0.0 | 0.0 |  |  |
| Land use | | | | |
| Urban | 3.08 | 3.11 | 0.99 | 0.36 |
| Non-urban | 0.0 | 0.0 |  |  |
| Rainfall (mm) | 0.03 | 0.02 | 1.27 | 0.21 |
| Human Population Density | 0.0 | 0.0 | 0.22 | 0.83 |
| Distance to Cover | 0.57 | 0.63 | 0.90 | 0.38 |
| Season | | | | |
| **Winter** | **3.08** | **1.15** | **2.68** | **<0.05** |
| Summer | 0.0 | 0.0 |  |  |
| *actm5 summary* | | | | |
| **Intercept** | **8.06** | **1.13** | **7.10** | **<0.001** |
| Land use | | | | |
| **Urban** | **3.26** | **1.50** | **2.18** | **0.05** |
| Non-urban | 0.0 | 0.0 |  |  |
| Season | | | | |
| **Winter** | **3.96** | **0.83** | **4.79** | **<0.001** |
| Summer | 0.0 | 0.0 |  |  |

| **Table S7:** Model selection table for the variables influencing the **number of vigilant acts**. Factors included in each model are denoted by the symbol –, an asterisk denotes interaction terms between variable. HPD (human population density), DTC (distance to cover). The full model and final model with the smallest AICc value are highlighted in bold, with these results being reported in full in table 8. | | | | | | | | | | | |
| --- | --- | --- | --- | --- | --- | --- | --- | --- | --- | --- | --- |
| **Model ID** | **intercept** | **Mob size** | **Sex** | **Land use** | **Rainfall (mm)** | **HPD** | **DTC** | **Season***  **Region** | **df** | **AICc** | **ΔAICc** |
| **a6** | **3.49** | **-** |  |  |  |  |  |  | **4** | **872.4** | **0.0** |
| a5 | 3.54 | - | - |  |  |  |  |  | 5 | 873.3 | 0.9 |
| a3 | 3.80 | - | - |  | - |  |  | - | 9 | 874.5 | 2.1 |
| a4 | 3.53 | - | - |  | - |  |  |  | 6 | 875.2 | 2.8 |
| a2 | 3.83 | - | - |  | - | - |  | - | 10 | 876.1 | 3.7 |
| a1 | 3.89 | - | - |  | - | - | - | - | 11 | 877.9 | 5.5 |
| **a0** | **3.87** | **-** | **-** | **-** | **-** | **-** | **-** | **-** | **12** | **879.8** | **7.4** |

| **Table S8:** Values from the LMM models highlighted in table 1 as being the final models used for predicting which factors influenced the **number of vigilant acts**. (Site was included as a random effect in each model, with the variance (s.d.) being 0.13 (0.37) in the full model and 0.10 (0.32) in model 6. Significant values are shown in bold. | | | | |
| --- | --- | --- | --- | --- |
| **Variable** | **Estimate** | **s.e.** | ***t­*-value** | ***p*-value** |
| *a0 summary* | | | | |
| **Intercept** | **3.87** | **2.60** | **14.87** | **<0.001** |
| Mob size | -0.09 | 0.05 | -1.92 | 0.06 |
| Sex | | | | |
| Male | -0.15 | 1.29 | -1.17 | 0.24 |
| Female | 0.0 | 0.0 |  |  |
| Land use | | | | |
| Urban | 0.05 | 0.32 | 0.15 | 0.88 |
| Non-urban | 0.0 | 0.0 |  |  |
| Rainfall (mm) | 0.0 | 0.0 | 0.74 | 0.46 |
| Human Population Density | 0.0 | 0.0 | -0.51 | 0.62 |
| Distance to Cover | -0.05 | 0.08 | -0.65 | 0.52 |
| Season | | | | |
| Winter | -0.30 | 0.18 | -1.65 | 0.10 |
| Summer | 0.0 | 0.0 |  |  |
| Region | | | | |
| ACT | -0.44 | 0.29 | -1.54 | 0.14 |
| SEQ | 0.0 | 0.0 |  |  |
| Season*Region | 0.26 | 0.28 | 0.93 | 0.35 |
| *a6 summary* | | | | |
| **Intercept** | **3.49** | **0.16** | **21.30** | **<0.001** |
| **Mob size** | **-0.11** | **0.04** | **-3.04** | **<0.05** |
